# Transcriptomic analysis of the oleaginous microalga *Neochloris oleoabundans* reveals metabolic insights into triacylglyceride accumulation

**DOI:** 10.1186/1754-6834-5-74

**Published:** 2012-09-24

**Authors:** Hamid Rismani-Yazdi, Berat Z Haznedaroglu, Carol Hsin, Jordan Peccia

**Affiliations:** 1 Department of Chemical and Environmental Engineering, Yale University, 9 Hillhouse Ave, New Haven, CT 06520, USA; 2 Department of Chemical Engineering, Massachusetts Institute of Technology, Cambridge, MA, 02139, USA

**Keywords:** Microalgae, Biofuel, *de novo* transcriptomics, Nitrogen limitation, RNA-Seq, Gene expression, Triacylglyceride, Triglyceride, *Neochloris oleoabundans*

## Abstract

**Background:**

The lack of sequenced genomes for oleaginous microalgae limits our understanding of the mechanisms these organisms utilize to become enriched in triglycerides. Here we report the *de novo* transcriptome assembly and quantitative gene expression analysis of the oleaginous microalga *Neochloris oleoabundans*, with a focus on the complex interaction of pathways associated with the production of the triacylglycerol (TAG) biofuel precursor.

**Results:**

After growth under nitrogen replete and nitrogen limiting conditions, we quantified the cellular content of major biomolecules including total lipids, triacylglycerides, starch, protein, and chlorophyll. Transcribed genes were sequenced, the transcriptome was assembled *de novo*, and the expression of major functional categories, relevant pathways, and important genes was quantified through the mapping of reads to the transcriptome. Over 87 million, 77 base pair high quality reads were produced on the Illumina HiSeq sequencing platform. Metabolite measurements supported by genes and pathway expression results indicated that under the nitrogen-limiting condition, carbon is partitioned toward triglyceride production, which increased fivefold over the nitrogen-replete control. In addition to the observed overexpression of the fatty acid synthesis pathway, TAG production during nitrogen limitation was bolstered by repression of the β-oxidation pathway, up-regulation of genes encoding for the pyruvate dehydrogenase complex which funnels acetyl-CoA to lipid biosynthesis, activation of the pentose phosphate pathway to supply reducing equivalents to inorganic nitrogen assimilation and fatty acid biosynthesis, and the up-regulation of lipases—presumably to reconstruct cell membranes in order to supply additional fatty acids for TAG biosynthesis.

**Conclusions:**

Our quantitative transcriptome study reveals a broad overview of how nitrogen stress results in excess TAG production in *N. oleoabundans*, and provides a variety of genetic engineering targets and strategies for focused efforts to improve the production rate and cellular content of biofuel precursors in oleaginous microalgae.

## Background

Important advantages of microalgae-based biofuels over first generation biofuels include algae’s greater solar energy conversion efficiency compared to land plants [1], the ability of oleaginous microalgae to utilize non-arable land and saline or waste-water, and their high content of energy dense neutral lipids that can be readily transesterified to produce biodiesel [2,3]. Under stress conditions such as nutrient deprivation or high light intensity, several species of oleaginous microalgae can alter lipid biosynthetic pathways to produce intracellular total lipid contents between 30 to 60% of dry cell weight (DCW) [4]. Triacylglycerides (TAGs) are the dominant form of lipids produced under these conditions. The excess production of TAGs in microalgae is thought to play a role in carbon and energy storage and functions as part of the cell’s stress response [5].

Due to the limited understanding of microalgae genetics and physiology, lipid metabolism from higher plants and bacteria have been the basis from which the accumulation of TAGs in microalgae has been modeled [5]. TAGs and polar membrane lipids are synthesized from fatty acids, that are primarily produced in the chloroplast [6]. The committed step in fatty acid biosynthesis starts with the conversion of acetyl CoA to malonyl CoA through the enzyme acetyl CoA carboxylase (ACCase). In some plants, there is evidence that both photosynthesis- and glycolysis-derived pyruvate could be endogenous sources of acetyl CoA pool for fatty acid biosynthesis [5]. Fatty acid production in *E. coli* is regulated through feedback-inhibition by long chain fatty acyl carrier proteins (ACP) [7,8], and a recent study in the microalgae *Phaeodactylum tricornutum* demonstrated that overexpression of genes that encode for the thioesterases that hydrolyze the thioester bond of long chain fatty acyl ACPs resulted in a significant increase in fatty acid production [9]. Recent nitrogen deprivation studies in the model, nonoleaginous microalga *Chlamydomonas reinhardtii* have also suggested an important role for lipases in restructuring the cell membrane under nitrogen limitation in order to supply fatty acids for TAG biosynthesis [10].

The stress-induced production of TAGs provides an opportunity to observe differential gene expression between high and low TAG accumulating phenotypes. Because multiple pathways are associated with the enhanced production of neutral lipids in microalgae, transcriptomic studies are an appropriate tool to provide an initial, broad view of carbon partitioning [11] and regulation of TAG biosynthesis during microalgae stress responses. However, the most promising strains thus far identified by growth experiments and lipid content screening [4,12] do not have sequenced, fully annotated genomes [13-15]. In microalgae, transcriptomic studies have instead focuses on model organisms that are not oleaginous but have sequenced genomes [10,16]. There is a growing number of oleaginous microalgae from which *de novo* transcriptomes have been assembled and annotated but comprehensive quantitative gene expression analysis in these microalgae has not yet been performed [14,17-19]. Recently, a *de novo* assembled-transcriptome was used as a search model to enable a proteomic analysis of the oleaginous microalga *Chlorella vulgaris* that demonstrated up-regulation of fatty acid and TAG biosynthetic pathways in response to nitrogen limitations [13].

In the present study, we quantitatively analyzed the transcriptome of the oleaginous microalga *Neochloris oleoabundans* to elucidate the metabolic pathway interactions and regulatory mechanisms involved in the accumulation of TAG. *N. oleoabundans* (a taxonomic synonym of *Ettlia oleoabundans*[20]) is a unicellular green microalga belonging to the *Chlorophyta* phylum (class *Chlorophyceae*). It is known to produce large quantities of lipids (35 to 55% dry cell weight total lipids and greater than 10% TAGs) [4,12,21] in response to physiological stresses caused by nitrogen deprivation. To produce differences in lipid enrichment, *N. oleoabundans* was cultured under nitrogen replete and nitrogen limited conditions and major biomolecules including total lipids, TAGs, starch, protein, and chlorophyll were measured. The transcriptome was sequenced and assembled *de novo*, gene expression was quantified, and comparative analysis of genes, pathways and broader gene ontology categories was conducted. The results provide new insight into the regulation of lipid metabolism in oleaginous microalgae at the transcriptomic level, and suggest several potential strategies to improve lipid production in microalgae based on a rational genetic engineering approach.

## Results

### Major biomolecule content and composition differ between the nitrogen replete (+N) and nitrogen-limited (−N) growth environments

**Figure 1:**
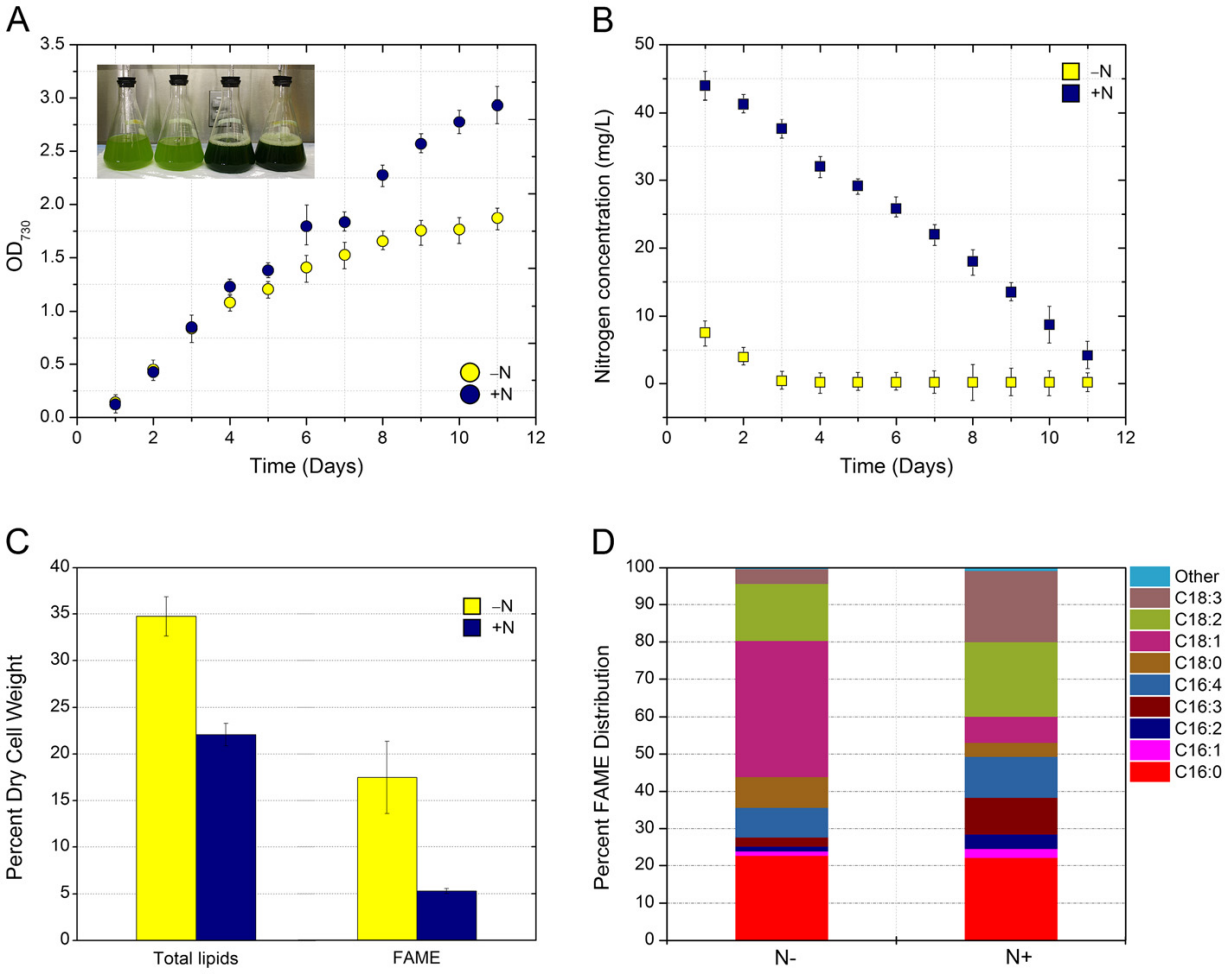
***N. oleoabundans *growth and lipid characteristics.** (**A**) Growth curves under + N and − N conditions. Inset image represents the difference in culture appearance between the two growth condition; (**B**) Nitrate as N concentrations in the bioreactors during growth; (**C**) Cell weight enrichment of total lipids and fatty acid methyl esters (FAME, representative of TAGs) from cells harvested on day 11; and (**D**) Percentage distribution of FAME from cells harvested on day 11. All error bars represent one standard deviation.

To track gene transcription in the oleaginous microalga *N. oleoabundans*, cells were first grown under + N and − N conditions as a method to produce differential cellular enrichments of TAGs. Cells were harvested after 11 days. This sampling time corresponded to below detection level concentrations for NO_3_^-^ and a reduction in growth rate in the –N reactors (Figure 1A, B). The maximum growth rate for the –N cultures was 113 ± 4 (std. err.) mgl^-1^ day^-1^ and decreased to 34 ± 0.7 mgl^-1^ day^-1^ once nitrogen became limited in the reactor. Total lipids extracted under the + N and − N scenarios revealed a statistically significant increase (*p* < 0.05) from 22% DCW in + N to 36% in the − N condition (Figure 1C). Extracted lipids were transesterified and fatty acid methyl esters (FAMEs) (FAMEs assumed to be equivalent to TAGs content [22]), were quantified. Compared to the + N condition, the FAME or TAG content per cell mass increased by five times in the − N case (*p* <0.05), demonstrating that the additional lipids produced during N limitations were mostly TAGs (Figure 1C). Estimates of total cell mass based on direct microscopic counts and DCW determinations revealed that the average mass of a cell in − N was 81% of that in + N, confirming that the change in TAG was independent of changes in DCW. FAME profiles are presented in Figure 1D, and show a 50% decrease in the proportion of unsaturated fatty acids (i.e. C16:2, C16:3, C16:4, C18:2, and C18:3) under nitrogen limitation. The most significant change was in the amount of oleic acid (C18:1), which increased over 5 times, while the quantity of α-linoleic acid (C18:3) decreased by 4.8-fold under –N conditions. This trend toward a greater proportion of C18:1 is consistent with prior investigations of the oleaginous microalgae *N. oleoabundans* and *Chlorella vulgaris* FAME contents under nitrogen limitations [13,22].

To aid in interpreting how photosynthetically fixed carbon was directed into major metabolic pathways, the chlorophyll, protein, and starch content of *N. oleoabundans* were also measured under the − N and + N scenarios (Table 1). Nitrogen deprivation lead to a reduction in nitrogen-containing chlorophyll content. This loss of chlorophyll is consistent with the light green color of chlorosis observed in the cultures under nitrogen limitation (Figure 1A inset). Also under nitrogen limitation, a decrease in cellular protein content and an increase in cellular starch content were observed. The observed changes in metabolite and biomolecule contents suggest the redirection of metabolism in *N. oleoabundans* during nitrogen limitation to reduce nitrogen-containing compounds (protein and chlorophyll) and favor the accumulation of nitrogen free storage molecules TAGs and starch.

**Table 1:** **Culture density and cellular composition of major biomolecules of *N. oleoabundans *cells determined after 11 days of growth under nitrogen replete (+N) and nitrogen limited (−N) conditions**

	**+N**	**–N**
Culture density (cells/mL)	(6.1 ± 0.2) × 10^7^	(3.8 ± 0.2) × 10^7^
Chlorophyll *a* (μg/mg)	(119.3 ± 12.6) × 10^-3^	(5.9 ± 0.4) × 10^-3^
Chlorophyll *b*(μg/mg)	(42.6 ± 5.5) × 10^-3^	(5.5 ± 0.5) × 10^-3^
Starch content (% DCW)	0.2 ± 0.1	4.0 ± 0.5
Protein content (% DCW)	37.9 ± 4.0	19.4 ± 17.1

### *De novo* transcriptome assembly, annotation, and expression

**Figure 2:**
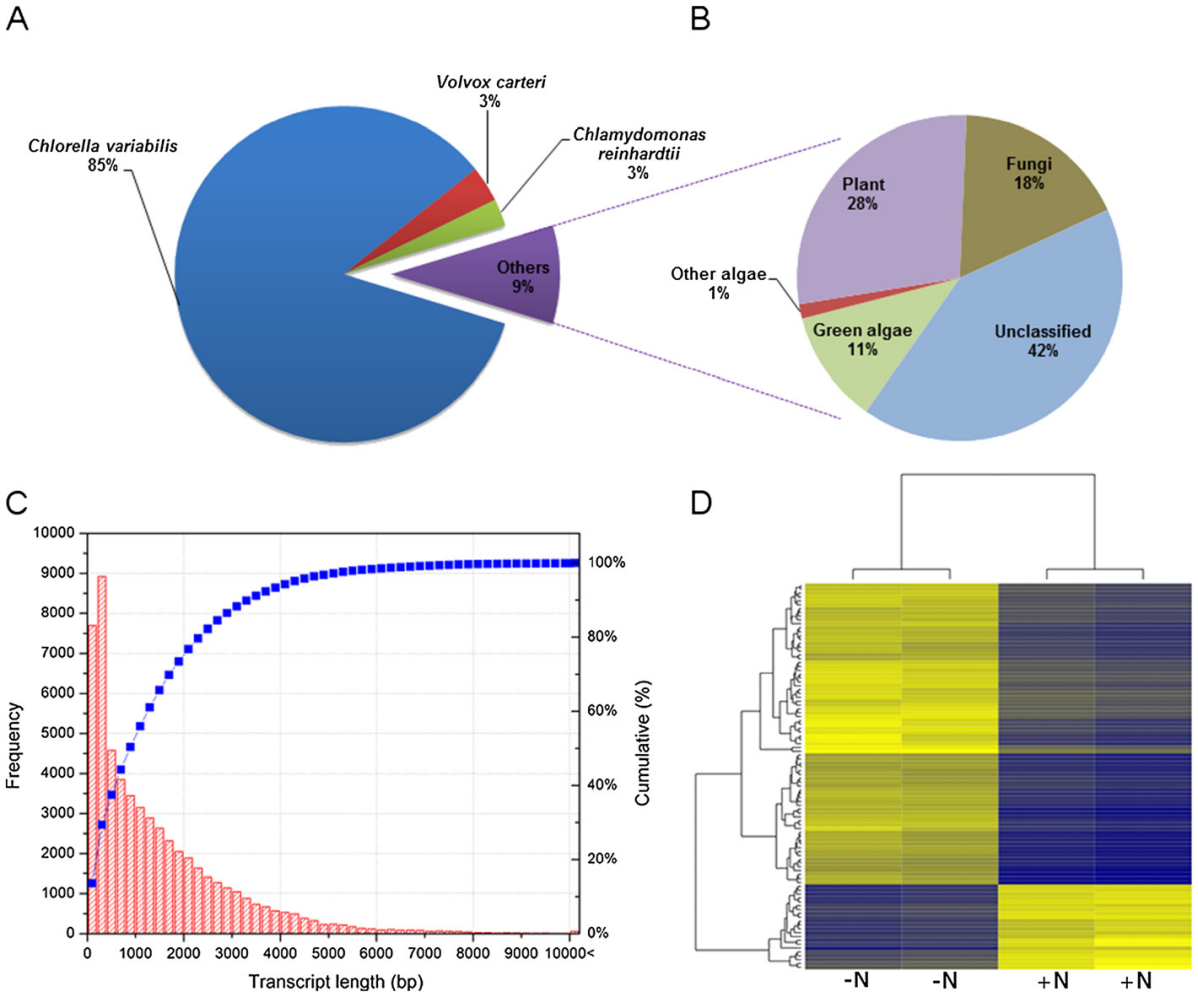
***De novo *assembly and mapping results.** (**A**, **B**) Top-hit species distribution for BLASTX matches for the *N. oleoabundans* transcriptome; (**C**) Cumulative transcript length frequency distribution of the *N. oleoabundans* transcriptome assembly; (**D**) Heat map demonstrating the top 100 most differentially expressed genes in the biological replicates of + N and − N conditions.

In order to produce statistically reliable and comparable RNA-Seq data, cDNA library construction and sequencing was performed for each of the duplicate + N reactors, and each of the duplicate − N reactors. Over 88 million raw sequencing reads were generated and subjected to quality score and length based trimming; resulting in a high quality (HQ) read data set of 87.09 million sequences (average phred score of 35) with an average read length of 77 bp (Additional file 1). By incorporating a multiple *k-*mer based *de novo* transcriptome assembly strategy (*k*-mers 23, 33, 63, and 83) [23], HQ reads were assembled into 56,550 transcripts with an average length of 1,459 bp and a read coverage of 1,444× (Additional file 1, Figure 2C). Generated transcripts were subjected to searches against the National Center for Biotechnology Information’s (NCBI) nonredundant and plant refseq databases [24], and the majority of transcripts showed significant mat-ches to other closely related green microalgae species (Figure 2A, B) including *C. variabilis* (~85% of all transcripts), *C. reinhardtii* (~2.6%), and *V. carteri* (~3.4%) (Figure 2A). With additional annotations by using KEGG services and Gene Ontology (GO), a total of 23,520 transcripts were associated with at least one GO term, and 4,667 transcripts were assigned with enzyme commission (EC) numbers. Overall, 14,957 transcripts had KO identifiers and were annotated as putative genes and protein families (see Additional file 2 for the annotation summary). This assembly provided a reliable, well-annotated transcriptome for downstream RNA-Seq data analysis.

**Figure 3:**
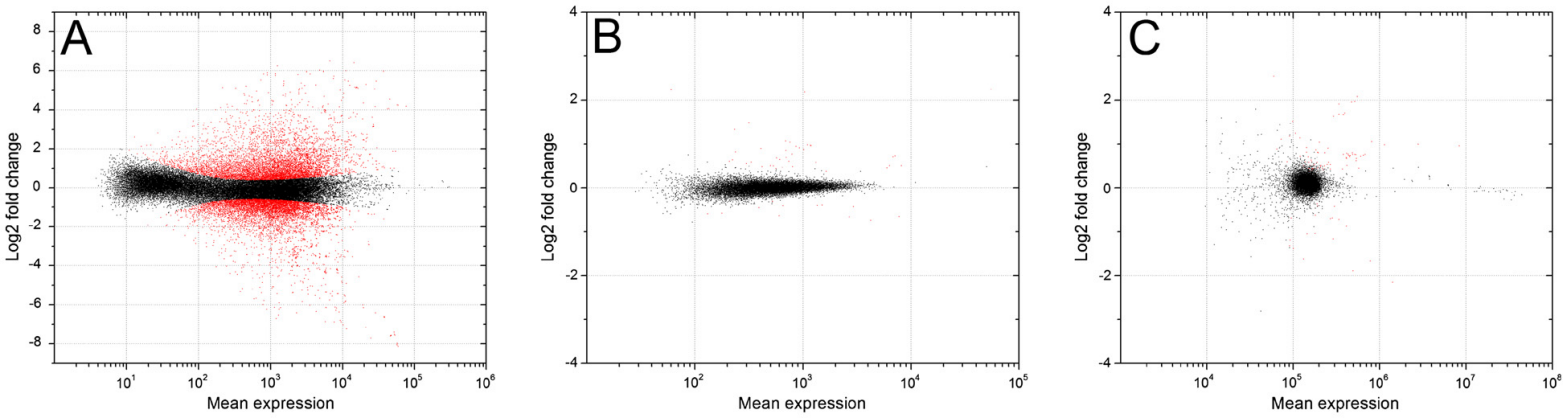
**(A) MvA plot contrasting gene expression levels between the − N and + N scenarios based on reads mapped to the*N. oleoabundans*transcriptome.** The x-axis represents the mean expression level at the gene scale, and the y-axis represents the log2 fold change from − N to + N. Negative fold changes indicate up-regulation of –N genes. Red dots are genes that are significant at a false discovery rate of 5%; (**B**) MvA plot for reads mapped to the *C. reinhardtii* genome; and (**C**) MvA plot for reads mapped to the *V. carteri* genome.

Following the transcriptome assembly and annotation, HQ reads obtained from each experimental condition were individually mapped to the generated assembly in order to determine the transcript abundances as RPKM values. To determine fold change differences among + N and − N transcripts, non-normalized read counts were fed into the DESeq package (v1.5.1) and variance and mean dependencies were accounted for [25]. Based on the negative binomial distribution model used in DESeq package, 25,896 transcripts out of the total 56,550 non-redundant transcripts were up-regulated under the − N condition. Plotting transcript fold changes levels shows a high correlation among the biologically replicated sequencing runs as indicated by Euclidean distances (Figure 2D). Overall, 15,987 transcripts had significant differential regulation (*q* < 0.05) Figure 3A. A complete table of fold changes with significance level for all genes assessed is presented in Additional file 3.

We further investigated the alignment of HQ reads to the reference genomes of *C. reinhardtii* and *V. carteri* in order to improve and extend our transcriptomic analysis to the detection of splicing events and alternative isoform formation (Figure 3B, C). Although the majority of annotated orthologs were identified from these closely related microalgae species, very poor mappings (i.e. <5% of reads) were observed between the RNA-Seq data of *N. oleoabundans* and the genomes of *C. reinhardtii* and *Volvox carteri.* As a result, the number of transcripts annotated and evaluated for differential expression was suboptimal, and genomes from these most closely related organisms were not used for gene expression analysis.

### Clustering of relevant GO terms and differential expression

**Figure 4:**
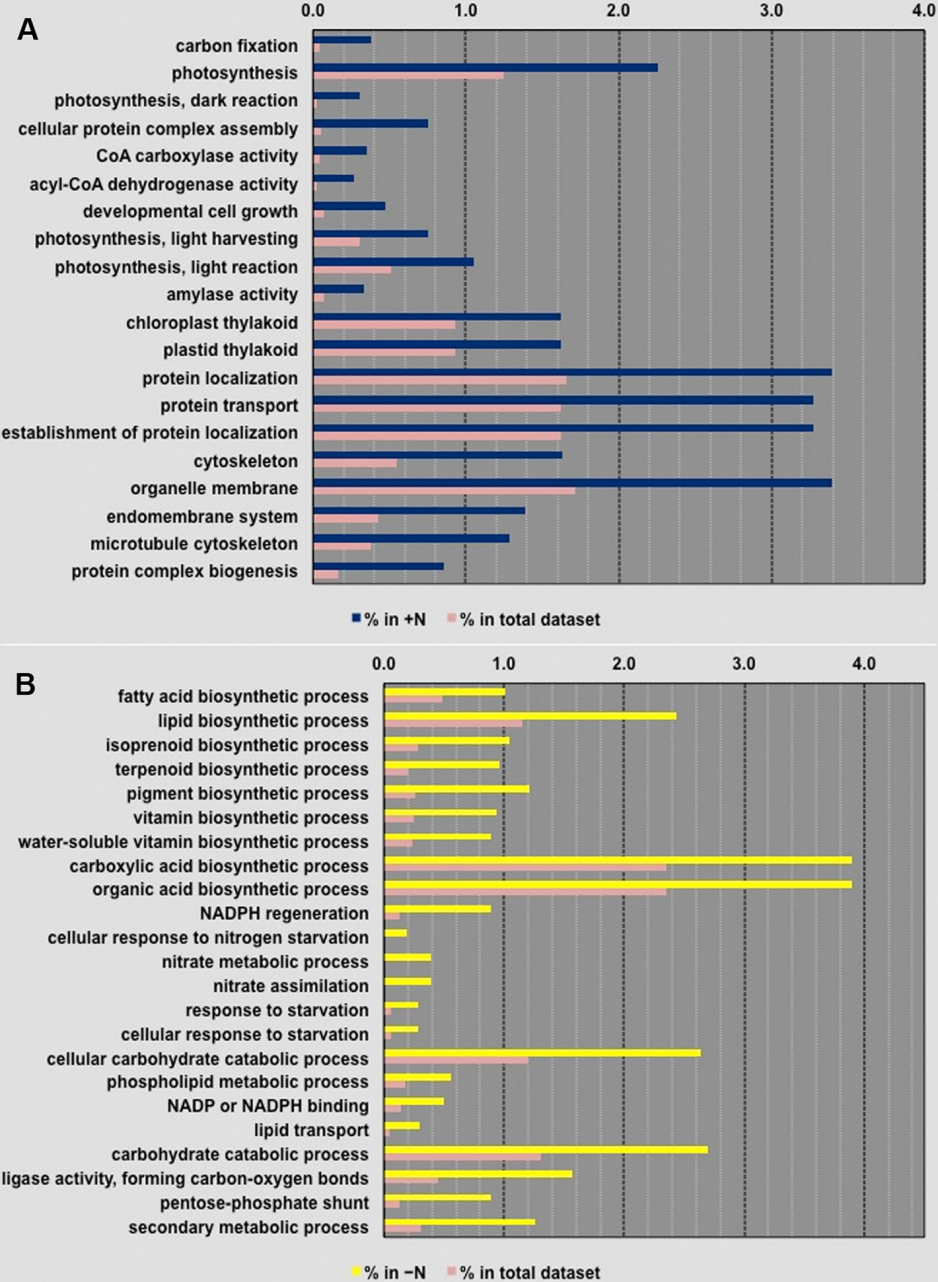
**Over representation analysis of selected significant GO terms.** (**A**) contains results for + N versus the full dataset and (**B**) contains results for − N versus the full dataset.

The transcripts annotated in + N and − N transcriptomes were first classified based on Gene Ontology (GO) terms. In the + N and –N datasets, respectively, 6,846 and 7,473 transcripts were classified into 306 and 218 broader GO term categories in accordance with the Gene Ontology Consortium [26] (Additional files 4 and 5). An enrichment analysis of the broader GO terms was performed using the modified Fisher’s Exact test in Blast2GO to quantitatively compare the distribution of differentially enriched GO terms between the + N case and the entire data set (Figure 4A), and between the –N case and the entire data set (Figure 4B). The functional categories enriched under + N were distinctly different from those enriched under the –N condition. In the + N case (Figure 4A), functional categories linked to carbon fixation, photosynthesis, protein machinery, and cellular growth were highly enriched compared to the –N condition; reflecting the higher growth rate, higher cell mass, and increased chlorophyll content observed in + N. Under –N conditions, genes associated with carboxylic acid and lipid biosynthetic process, NADPH regeneration, the pentose-phosphate pathway, phospholipid metabolic process, and lipid transport demonstrated a greater enrichment of transcripts than the overall dataset (Figure 4B). These enriched GO terms directly correlated with the observed increase of lipid accumulation in –N cells. Other major categories identified as significantly expressed under the –N condition included the synthesis of value added products such as terpenoids, pigments, and vitamins as well as cellular response to nitrogen starvation, nitrate metabolic process, and nitrate assimilation (Figure 4B). Genes involved in the latter three functional categories were exclusively expressed in the nitrogen-limited cells.

### Fatty acid biosynthesis pathway is up-regulated and the β-oxidation pathway is repressed under nitrogen-limiting conditions

**Figure 5:**
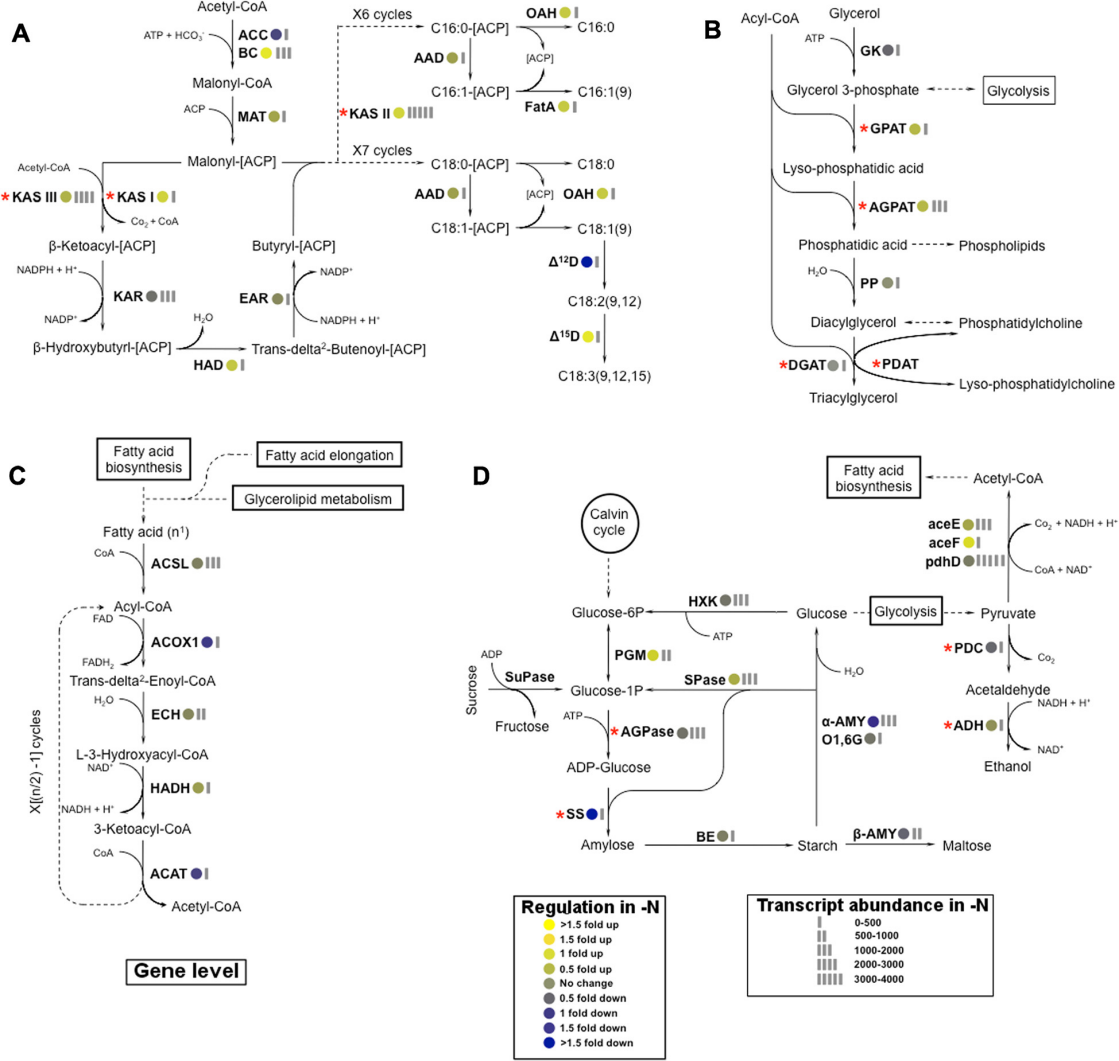
**Differential expression of genes involved in (A) the fatty acid biosynthesis; (B) triacylglycerol biosynthesis; (C)β-oxidation; and (D) starch biosynthesis.** Pathway were reconstructed based on the *de novo* assembly and quantitative annotation of the *N. oleoabundans* transcriptome. (**A**) Enzymes include: ACC, acetyl-CoA carboxylase (EC: 6.4.1.2); MAT, malonyl-CoA ACP transacylase (EC: 2.3.1.39); KAS, beta-ketoacyl-ACP synthase (KAS I, EC: 2.3.1.41; KASII, EC: 2.3.1.179; KAS III, EC: 2.3.1.180); KAR, beta-ketoacyl-ACP reductase (EC: 1.1.1.100); HAD, beta-hydroxyacyl-ACP dehydrase (EC: 4.2.1.-); EAR, enoyl-ACP reductase (EC: 1.3.1.9); AAD, acyl-ACP desaturase (EC: 1.14.19.2); OAH, oleoyl-ACP hydrolase (EC: 3.1.2.14); FatA, Acyl-ACP thioesterase A (EC: 3.1.2.-); Δ^12^D, Δ^12^(ω^6^)-desaturase (EC: 1.4.19.6); Δ^15^D, Δ^15^(ω^3^)-desaturase (EC: 1.4.19.-); (**B**) Enzymes include: GK, glycerol kinase (EC: 2.7.1.30); GPAT, glycerol-3-phosphate *O*-acyltransferase (EC: 2.3.1.15); AGPAT, 1-acyl-sn-glycerol-3-phosphate *O*-acyltransferase (EC:2.3.1.51); PP, phosphatidate phosphatase (EC: 3.1.3.4); DGAT, diacylglycerol *O*-acyltransferase (EC: 2.3.1.20); and PDAT, phopholipid:diacyglycerol acyltransferase (EC 2.3.1.158); (**C**) Enzymes include: ACS, acyl-CoA synthetase (EC: 6.2.1.3); ACOX1, acyl-CoA oxidase (EC: 1.3.3.6); ECH, enoly-CoA hydratase (EC: 4.2.1.17); HADH, 3-hydroxyacyl-CoA dehydrogenase (EC: 1.1.1.35); ACAT, acetyl-CoA *C*-acetyltransferase (EC: 2.3.1.16, 2.3.1.9); (**D**) Enzymes include: PGM, phosphoglucomutase (EC: 5.4.2.2); AGPase, ADP-glucose pyrophosphorylase (EC: 2.7.7.27); SS, starch synthase (EC: 2.4.1.21); BE, α-1,4-glucan branching enzyme (EC: 2.4.1.18); and HXK, hexokinase (2.7.1.1). Starch catabolism enzymes include: α-AMY, α-amylase (EC: 3.2.1.1); O1,6G, oligo-1,6-glucosidase (EC: 3.2.1.10); β-AMY, β-amylase (EC: 3.2.1.2); and SPase, starch phosphorylase (EC: 2.4.1.1). Ethanol fermentation via pyruvate enzymes include: PDC, pyruvate decarboxylase (EC: 4.1.1.1); and ADH, alcohol dehydrogenase (EC: 1.1.1.1). Enzymes aceE, pyruvate dehydrogenase E1 component (EC 1.2.4.1); aceF, pyruvate dehydrogenase E2 component (EC: 2.3.1.12); and pdhD, dihydrolipoamide dehydrogenase (EC 1.8.1.4), transforms pyruvate into acetyl-CoA. Key enzymes are shown with an asterisk (*) next to the enzyme abbreviations, and dashed arrows denote reaction(s) for which the enzymes are not shown. All presented fold changes are statistically significant, q value < 0.05.

The majority of genes governing fatty acid biosynthesis were identified as being overexpressed in nitrogen limited cells as shown in the global metabolic pathway level and fatty acid biosynthesis module (Additional file 6). The fold-change and abundances of identified transcripts for the components of fatty acid biosynthesis at the gene level are presented in Figure 5A. The first step in fatty acid biosynthesis is the transduction of acetyl-CoA into malonyl-CoA by addition of carbon dioxide. This reaction is the first committing step in the pathway and catalyzed by Acetyl-CoA Carboxylase (ACCase). While the gene encoding ACCase was repressed under the –N condition, the biotin-containing subunit of ACCase, biotin carboxylase (BC), was significantly up-regulated in response to nitrogen starvation. The BC catalyzes the ATP-dependent carboxylation of the biotin subunit and is part of the heteromeric ACCase that is present in the plastid—the site of *de novo* fatty acid biosynthesis [27]. To proceed with fatty acid biosynthesis, malonyl-CoA is transferred to an acyl-carrier protein (ACP), by the action of malonyl-CoA ACP transacylase (MAT). This step is followed by a round of condensation, reduction, dehydration, and again reduction reactions catalyzed by beta-ketoacyl-ACP synthase (KAS), beta-ketoacyl-ACP reductase (KAR), beta-hydroxyacyl-ACP dehydrase (HAD), and enoyl-ACP reductase (EAR), respectively. The expression of genes coding for MAT, KAS, HAD, and EAR were up-regulated, whereas the KAR encoding gene was repressed in –N cells. The synthesis ceases after six or seven cycles when the number of carbon atoms reaches sixteen (C16:0-[ACP]) or eighteen (C18:0-[ACP]). ACP residues are then cleaved off by thioesterases oleoyl-ACP hydrolase (OAH) and Acyl-ACP thioesterase A (FatA) generating the end products of fatty acid synthesis (i.e. palmitic (C16:0) and stearic (C18:0) acids). Genes coding for these thioesterases, i.e. FatA and OAH, were overexpressed in –N cells. The up-regulation of these thioesterase encoding genes, as previously reported in *E. coli* and the microalga *P. tricornutum*, is associated with reducing the feedback inhibition that partially controls the production rate of fatty acid biosynthesis [7,8], and results in the overproduction of fatty acids [9]. It has also been suggested that an increase in FatA gene expression and the associated acyl-ACP hydrolysis may aid in increased fatty acid transport from the chloroplast to the endoplasmic reticulum site where TAG assembly occurs [10,28]. Finally, for supplying reducing equivalents via NADPH to power fatty acid biosynthesis, genes encoding for the pentose phosphate pathway were strongly up-regulated in the –N condition (Table 2).

**Table 2:** ***N. oleoabundans *genes involved in the pentose phosphate pathway**

**Pentose phosphate pathway**	**Log2FC**
Phosphogluconate dehydrogenase (decarboxylating) (PGD, EC: 1.1.1.44)	−1.13
Glucose-6-phosphate dehydrogenase (G6PD, EC: 1.1.1.49)	−1.41
Transketolase (tktA, EC: 2.2.1.1)	2.55
Transaldolase (talA, EC: 2.2.1.2)	−0.66
6-phosphofructokinase (PFK, EC: 2.7.1.11)	−0.45
Gluconokinase (gntK, EC: 2.7.1.12)	0.10
Ribokinase (rbsK, EC: 2.7.1.15)	0.11
Ribose-phosphate diphosphokinase (PRPS, EC: 2.7.6.1)	−0.10
Gluconolactonase (GNL, EC: 3.1.1.17)	−0.67
6-phosphogluconolactonase (PGLS, EC: 3.1.1.31)	0.07
Fructose-bisphosphatase (FBP, EC: 3.1.3.11)	−0.24
Fructose-bisphosphate aldolase (fbaB, EC: 4.1.2.13)	0.17
Ribulose-phosphate 3-epimerase (RPE, EC: 5.1.3.1)	−0.11
Ribose-5-phosphate isomerase (rpiA, EC: 5.3.1.6)	−0.34
Glucose-6-phosphate isomerase (GPI, EC: 5.3.1.9)	−1.21
Phosphoglucomutase (pgm, EC: 5.4.2.2)	−0.83

Negative Log2FC values represent up-regulation under nitrogen limitation. All presented fold changes are statistically significant, q value < 0.05.

The altered expression of genes associated with the generation of double bonds in fatty acids reflects the observed increase in the proportion of unsaturated of fatty acids (Figure 1D), and the enrichment of C18:1 during nitrogen limitations. The acyl-ACP desaturase (AAD), which introduces a one double bond to C16:0/C18:0, and delta-15 desaturase, which converts C18:2 to C18:3, were significantly up-regulated in the − N case, whereas the delta-12 desaturase catalyzing the formation of C18:2 from C18:1was repressed during nitrogen limitation.

Under nitrogen limitations, 10 of the 13 genes associated with fatty acid degradation (α and β-oxidation pathways for saturated and unsaturated acids) were significantly repressed. Figure 5C demonstrates the typical β-oxidation pathway for saturated fatty acids, while Table 3 displays expression levels for additional peroxisomal genes associated with fatty acid oxidation, but not shown in Figure 5C. Before undergoing oxidative degradation, fatty acids are activated through esterification to Coenzyme A. The activation reaction, is catalyzed by acyl-CoA synthetase (ACSL), which was up-regulated in –N cells. The acyl-CoA enters the β-oxidation pathway and undergoes four enzymatic reactions in multiple rounds. The first three steps of the pathway; oxidation, hydration and again oxidation of acyl-CoA are catalyzed by acyl-CoA oxidase (ACOX1), enoly-CoA hydratase (ECH), and hydroxyacyl-CoA dehydrogenase (HADH), respectively. In the last step of the pathway, acetyl-CoA acetyltransferase (ACAT) catalyzes the cleavage of one acetyl-CoA, yielding a fatty acyl-CoA that is 2 carbons shorter than the original acyl-CoA. The cycle continues until all the carbons are released as acetyl-CoA. The expression level of ECH and HADH were unchanged and genes encoding for enzymes ACOX1 and ACAT catalyzing the first and last reactions in the cycle were identified as significantly repressed in − N cells.

**Table 3:** ***N. oleoabundans *genes involved in catabolic pathways related to peroxisomal fatty acid oxidation, lysosomal lipases, and the regulation of autophagy**

**Enzyme encoding gene**	**Log2FC**
Peroxisome	
*α-oxidation*	
2-hydroxyacyl-coa lyase 1 (HACL1, EC: 4.1.-.-)	0.35
*Unsaturated fatty acid β-oxidation*	
Peroxisomal 2,4-dienoyl-coa reductase (DECR2, EC: 1.3.1.34)	0.21
Δ(3,5)-Δ(2,4)-dienoyl-coa isomerase (ECH1, EC: 5.3.3.-)	−0.27
ATP-binding cassette, subfamily D (ALD), member 1 (ABCD1)	0.25
Long-chain acyl-coa synthetase (ACSL, EC: 6.2.1.3)	0.25
*Other oxidation*	
Peroxisomal 3,2-trans-enoyl-coa isomerase (PECI, EC: 5.3.3.8)	0.59
Carnitine O-acetyltransferase (CRAT, EC: 2.3.1.7)	0.30
NAD + diphosphatase (NUDT12, EC: 3.6.1.22)	0.47
Glycerolipid metabolism	
Triacylglycerol lipase (EC: 3.1.1.3)	0.33
Acylglycerol lipase (MGLL, EC: 3.1.1.23)	−0.13
Glycerophospholipid metabolism	
Phospholipase A1 (plda, EC: 3.1.1.32)	−1.26
Phospholipase A2 (PLA2G, EC: 3.1.1.4)	−0.31
Phospholipase C (plcc, EC: 3.1.4.3)	−0.10
Lysosome	
*Lipases*	
Lysosomal acid lipase (LIPA, EC: 3.1.1.13)	−0.48
Lysophospholipase III (LYPLA3, EC: 3.1.1.5)	0.20
Regulation of autophagy	
Unc51-like kinase (ATG1, EC: 2.7.11.1)	−0.53
5'-AMP-activated protein kinase, catalytic alpha subunit (snrk1, PRKAA)	−0.05
Vacuolar protein 8 (VAC8)	0.13
Beclin 1 (BECN1)	−0.59
Phosphatidylinositol 3-kinase (VPS34, EC: 2.7.1.137)	−1.26
Phosphoinositide-3-kinase, regulatory subunit 4, p150 (VPS15, EC: 2.7.11.1)	0.11
Autophagy-related protein 3 (ATG3)	0.11
Autophagy-related protein 4 (ATG4)	−0.16
Autophagy-related protein 5 (ATG5)	−0.27
Autophagy-related protein 7 (ATG7)	0.17
Autophagy-related protein 8 (ATG8)	−0.50
Autophagy-related protein 12 (ATG12)	−0.58

Negative log2 fold change (Log2FC) values represent up-regulation under nitrogen limitation. All presented fold changes are statistically significant, q value < 0.05.

### Nitrogen limitation and the regulation of genes associated with TAG biosynthesis

TAG is the major storage lipid in oleaginous microalgae and in this study nitrogen limitations induced a five-fold increase in its intracellular content. Several genes involved in TAG biosynthesis displayed changes in their expression in response to nitrogen limitation. Biosynthesis of TAG in the chloroplast begins with two consecutive acyl transfers from acyl-CoA to positions 1 and 2 of glycerol-3-phosphate to form phosphatidic acid (PA), which is subsequently dephosphorylated to form 1,2-diacylglycerol (DAG) (Figure 5B). These reactions are catalyzed by enzymes glycerol-3-phosphate acyltransferase (GPAT), acyl-glycerol-3-phosphate acyltransferase (AGPAT), and phosphatidate phosphatase (PP), respectively. The last step in the pathway, catalyzed by diacylglycerol acyltransferase (DGAT), involves the transfer of third acyl group to the DAG 3 position. This final reaction is the only dedicated step in TAG synthesis since the preceding intermediates (i.e. PA and DAG), are also substrates for the synthesis of membrane lipids. Our results indicated that the expression of genes encoding GPAT and AGPAT was up-regulated in response to nitrogen starvation. However, the expression of gene encoding PP and DGAT remained relatively unchanged.

Though TAG biosynthesis in microalgae is believed to occur mainly through the glycerol pathway as described above, an alternative route known as the acyl CoA-independent mechanism has also been reported to take place in some plants and yeast [29]. In this mechanism, phospholipid is utilized as the acyl donor in the last step of TAG formation and the reaction is catalyzed by phospholipid:diacylglycerol acyltransferase (PDAT). We have recently found homologues of gene encoding for PDAT in the *D. tertiolecta* transcriptome, suggesting that the PDAT route could also play a role in microalgae TAG biosynthesis [14]. We did not however identify such homologues in the transcriptome of *N. oleoabundans*, making it unclear if PDAT contributes to TAG biosynthesis in this organism.

### During nitrogen limitation genes associated with lipases and regulating autophagy are up-regulated

All three phospholipases encoding genes identified were overexpressed in –N, while only one of the two TAG lipase genes found, acylglycerol lipase, was overexpressed (Table 3). The overexpression of lipase genes during nitrogen deprivation in *C. reinhardtii* has been thought to be associated with the reconstruction of the cellular membrane for the purpose of channeling fatty acids to triacylglyceride production [10]. Triacylglyceride lipase, which is active in triacylglyceride hydrolysis was moderately repressed (log2 fold change = 0.33) under the –N scenario providing some support to the hypothesis that while membrane reconstruction was active, TAG degradation was reduced under nitrogen limitation (Table 3). Finally, genes associated with regulating autophagy and the 5’ AMP-activated protein kinase gene (SnRK1 gene in plants) were overexpressed in the − N scenario (Table 3). SnRK1 is a global regulator of carbon metabolism in plants [30,31], and its up regulation—along with that of autophagy associated genes—further demonstrates the cells efforts to maintain homeostasis under –N conditions.

### Nitrogen limitation affects the nitrogen-assimilatory pathway at the transcriptome level

We identified a number of genes that encode for components of the nitrogen assimilatory pathway (Table 4, Additional file 6, C). Genes that encode for enzymes catalyzing the reduction of NO_3_^-^ to NH_4_^+^ and the biosynthesis of nitrogen-carrying amino acids were strongly expressed under nitrogen limitation [32,33]. Along with the pentose phosphate pathway, these genes were among the most up-regulated genes in –N cells of *N. oleoabundans*. The increased expression of these genes was consistent with their role in nitrogen uptake and assimilation, and the nitrogen limited growth environment from which cells were derived.

**Table 4:** ***N. oleoabundans *genes involved in nitrogen assimilation**

**Nitrogen assimilation**	**Log2FC**
High affinity nitrate transporters	−4.4
Ammonium transporters	−2.8
Nitrate reductase (NR, EC: 1.7.1.1)	−3.8
Ferredoxin-nitrite reductase (NiR, EC: 1.7.7.1)	−3.9
Glutamine synthetase (GS, EC: 6.3.1.2)	−2.3
Glutamate synthase (NADH) (GOGAT, EC: 1.4.1.13-14)	−1.4
Glutamate synthase (Ferredoxin) (EC: 1.4.7.1)	0.27
Glutamate dehydrogenase (GDH, EC: 1.4.1.3)	0.89
Aspartate aminotransferase (aspat, EC: 2.6.1.1)	−2.3
Asparagine synthetase (AS, EC: 6.3.5.4)	−1.5

Negative Log2FC values represent up-regulation under nitrogen limitation. All presented fold changes are statistically significant, q value < 0.05.

### Starch synthesis under nitrogen limitations

While several genes associated with the preparatory steps in starch synthesis are up-regulated in the –N case, the genes encoding for key enzymes AGPase and starch synthase were repressed ((Additional file 6, B), 5D, Table 1). The degradative side of starch metabolism, specifically α-amylase which hydrolyzes starch to glucose, was also strongly repressed during nitrogen limitations. When coupled to the increased but still overall low starch contents in the –N case (Table 3), these findings suggest that the –N cells accumulated starch by repressing starch degradation. From (Additional file 6, B), it is also notable that pyruvate kinase (log2FC = −0.21) and the three-enzyme pyruvate dehydrogenase complex for converting glucose to acetyl-CoA (to supply fatty acid synthesis) were up-regulated during nitrogen limitation (Figure 5D).

## Discussion

Oleaginous microalgae can accumulate large quantities of lipid under stress inducing growth conditions, making them a target organism for sustainable liquid biofuel production. In the present study, we induced TAG production and accumulation in *N. oleoabundans* through nitrogen deprivation, and investigated the expression of genes involved in TAG production at the transcriptome level. Mapping reads to the assembled and annotated transcriptome provided significantly more information than mapping reads to other microalgae for which the genome has been sequenced and annotated (Figure 3). While transcriptomic analysis is not substitute for detailed gene and pathway studies, it does provide a broad overview of the important metabolic processes from which to efficiently build hypotheses that can guide future detailed studies on improving lipid accumulation.

Our results suggest that under –N conditions, the altered expression of coordinated metabolic processes, many of which occur in the plastid, redirect the flow of fixed carbon toward biosynthesis and storage of lipids. These processes include up-regulation of *de novo* fatty acid and TAG synthesis, and concomitant repression of β-oxidation and TAG lipases. To supply precursors for lipid production, genes associated with the pyruvate dehydrogenase complex for converting pyruvate to acetyl CoA and lipases involved in the release free fatty acids from cell wall glycerophospholipids were overexpressed in the –N scenario. To power fatty acid production, strong overexpression under –N was observed in the pentose-phosphate pathway, which is primarily involved in supplying reducing equivalents for anabolic metabolism, including the production of fatty acids and assimilation of inorganic nitrogen [34].

### Transcriptome response of *N. oleoabundans* to nitrogen limitation

A primary physiological response to nitrogen limitation is a decrease in cell growth, as observed with the three times reduction in *N. oleoabundans* growth rate. The transcript profile of nitrogen-starved *N. oleoabundans* clearly reflects the decrease in cell proliferation and stressed physiological status of the cells. Gene ontology terms related to cellular growth, photosynthesis, and protein machinery are significantly suppressed under –N conditions, and autophagy genes were up-regulated. The 5’ AMP-activated protein kinase (SnRK1 gene in plants) was slightly overexpressed in the − N scenario. SnRK1 is activated under starvation conditions, including nitrogen depletion [31] and is a global regulator of starch and TAGs production in plants [30]. Overexpression of SnRK1 in the transgenic potato *Solanum tubersum* cv. Prairie [35] and *Arabidopsis thaliana*[36] has resulted in changes in starch and carbohydrate levels, thus confirming this gene’s central role in carbon partitioning and suggesting that SnRK1 may be an important target for metabolic engineering efforts in oleaginous microalgae. We note also that genes encoding for the components of nitrogen assimilation are identified as the most significantly up-regulated genes in the transcriptome of nitrogen limited *N. oleoabundans*. Overexpression of nitrogen assimilation pathways under nitrogen limiting conditions has been previously reported in the transcriptome of other non-oleaginous microalgae species [10,33].

### The regulation of fatty acid and TAG biosynthesis and supply of precursors

While under nitrogen deprivations, there has been considerable uncertainty expressed whether the increase in TAG content is due to a reduction in the mass of the cell, rather than increase in TAG production [2]. Both the measured increase in TAG content per cell dry weight reported here (which accounted for the loss of cell mass during nitrogen limitation), and the observed changes in the FAME profile unequivocally demonstrate the overproduction and accumulation of TAG in *N. oleoabundans* under nitrogen stress. Quantitative gene expression results also support these TAG production observations. In our study, most of the genes involved in the fatty acid biosynthetic pathway were up-regulated under –N conditions. The gene encoding for ACCase, the first enzyme in the pathway, was reported as down-regulated under –N. However, the biotin-containing subunit of ACCase, biotin carboxylase (BC), was significantly overexpressed. In photosynthetic organisms, two different forms of ACCase have been identified, one located in the plastid and the other located in the cytosol. The plastidal ACCase is a heteromeric multi-subunit enzyme that contains BC, whereas the cytosolic ACCase is a homomeric multifunctional protein that does not contain BC [27]. In our transcriptome analysis, we identified genes encoding for both forms of ACCase. In the plastid—the primary cite of lipid biosynthesis in microalgae—we have observed a significant increase in expression of the BC subunit of heteromeric isoform that catalyzes the very first step of carboxylation. On the other hand, the expression of homomeric ACCase, predominantly located in the cytosol where lipid biosynthesis does not typically occur, was repressed.

Although the overexpression of BC points to a key step in the pathway as a potential target to genetically engineer an improved oleaginous strain, mixed results for improving fatty acid synthesis in microalgae have been observed when ACCase is overexpressed [2]. Recent research has suggested that fatty acid synthesis may also be regulated by inhibition from the buildup of long chain fatty acyl ACPs [9]. Overexpressing genes that cleave ACP residues from the long chain fatty acyl ACPs is a condition observed in bacteria and recently in the microalga *P. tricornutum* to result in increased production of fatty acids [9]. In our study, genes encoding for these enzymes were highly overexpressed under the –N conditions. Therefore, a potential target for metabolic engineering in *N. oleoabundans* is the overexpression of thioesterases FatA and OAH that cleave off ACP residues.

Genes encoding enzymes involved in the steps downstream of fatty acid biosynthesis, including elongation and desaturation, have also displayed significant changes in transcription levels in response to nitrogen starvation. In particular, the genes encoding AAD and delta-15 desaturase, which catalyze the formation of double bond between the 9^th^, 10^th^, 14^th^, and 15^th^ carbon, respectively, were up-regulated under –N conditions. A similar observation has been reported by Morin et al. [37], where the gene encoding delta-9 fatty acid desaturase is up-regulated in the oleaginous yeast *Y. lipolytica* cultured under nitrogen limitation. As observed here, and supported by gene expression levels, nitrogen limitation alter the lipid profile towards higher saturation (increase in C18:1, and decrease in C18:2 and C18:3). The increased proportion of saturated fatty acids in TAG has been demonstrated to improve cetane number and stability of resulting biodiesel [38].

Based on the lipid metabolism genes discovered from our transcriptome assembly, the acyl-CoA dependent mechanism is the major contributor to TAG biosynthesis in *N. oleoabundans*. In our study, two genes associated with biosynthesis of TAG show significant changes in their expression under –N condition: one encoding GPAT and the other one encoding AGPAT. These enzymes catalyze the acyl-CoA-dependent acylation of positions 1 and 2 of glycerol-3-phosphate, respectively. The acylation of glycerol-3-phosphate represents the first and committed step in glycerolipid biosynthesis, and likely the rate limiting step in the pathway as GPAT exhibits the lowest specific activity among all enzymes involved in the glycerol-3-phosphate pathway [39]. A recent proteomics study also reported significant up-regulation of TAG-related acyltransferases in parallel with accumulation of large quantities of lipid in *C. vulgaris* cultured under nitrogen limitation [13]. The overexpression of GPAT and AGPAT has been reported to increase seed oil accumulation in *Arabidopsis* and *Brassica napus*[40-42]. The up-regulation of these two genes also indicates an increase in the flow of acyl-CoA toward TAG biosynthesis. The final step of the TAG biosynthesis pathway is catalyzed by DGAT, the third acyltransferase. In our study, the gene encoding DGAT displays relatively no change in its expression under nitrogen limitation. This observation coupled with the significant increase in TAG production in the –N case, and previous proteomics studies that showed overexpression of DGAT in the *C. vulgaris* due to nitrogen limitation [13] provides evidence that DGAT expression in *N. oleoabundans* may be regulated post-transcriptionally. The post-transcriptional regulation of DGAT has previously been documented in the oilseed rape *Brassica napus*[43].

Finally, the enrichment of intracellular starch increased during the –N case. Although starch synthase and AGPase encoding genes were repressed in –N, the gene encoding for α-amylase, responsible for the hydrolysis of starch to glucose monomers, was also repressed. The concomitant accumulation of starch and lipids under nitrogen limitation has been reported in the nonoleaginous *C. reinhardtii*[44,45] and recently reported for *N. oleoabundans*[46]. This contrasts with recent reports in *Micractinium pusillum* where carbohydrate content was reduced and TAG production was increased under nitrogen limitation [19]. Genetic manipulations (sta6 mutant) that block starch synthesis in *C. reinhardtii* have resulted in a significant increase in TAG accumulation [47]. Under nitrogen limitation, the increased TAG content in *N. oleoabundans* and concomitant repression of starch synthase are analogous to the *C. reinhardtii* sta6 mutant. These results extend the idea of blocking starch synthesis for improvement of TAG production to the oleagenous microalga *N. oleoabundans*.

### Lipid turnover

In our study, several genes encoding enzymes involved in the intracellular breakdown of fatty acids and lipids are significantly repressed under –N (Table 3). Repressing β-oxidation is a clear strategy for maintaining a higher concentration of fatty acids within a cell. In contrast, most of the identified lipases (with the exception of triacylglycerol lipases) are overexpressed during nitrogen limitation. Upon closer examination, the up-regulated lipases are mostly phospholipases associated with hydrolyzing cell wall glycerophospholipids and phospholipids into free fatty acids, potentially for incorporation into TAGs. A known result of nitrogen limitation induced autophagy in *C. reinhardtii* is the degradation of the chloroplast phospholipid membrane [47,48]. Moreover, the overexpression of lipases during nitrogen limitation in *C. reinhardtii* has previously been hypothesized to be associated with the reconstruction of cell membranes [10]. In addition to phospholipases, we have identified an enriched number of transcripts for phospholipid metabolic processes and lipid transport in the –N case (Figure 4B). The up-regulation of genes encoding for enzymes that produce free fatty acids is also consistent with the fact that the PDAT enzyme associated with the acyl-CoA-independent mechanism of TAG synthesis (which utilizes phospholipids, rather than free fatty acids, as acyl donors) was not recovered in our assembled transcriptome.

## Conclusions

Assembling the transcriptome and quantifying gene expression responses of *Neochloris oleoabundans* under nitrogen replete and nitrogen limited conditions enabled the exploration of a broad diversity of genes and pathways, many of which comprise the metabolic responses associated with lipid production and carbon partitioning. The high coverage of genes encoding for full central metabolic pathways demonstrates the completeness of the transcriptome assembly and the repeatability of gene expression data. Furthermore, the concordance of metabolite measurements and observed physiological responses with gene expression results lends strength to the quality of the assembly and our quantitative assessment. Our findings point to several molecular mechanisms that potentially drive the overproduction of TAG during nitrogen limitation. These include up-regulation of fatty acid and TAG biosynthesis associated genes, shuttling excess acetyl CoA to lipid production through the pyruvate dehydrogenase complex, the role of autophagy and lipases for supplying an additional pool of fatty acids for TAG synthesis, and up-regulation of the pentose phosphate pathway to produce NADPH to power lipid biosynthesis. These identified gene sequences and measured metabolic responses during excess TAG production can be leveraged in future metabolic engineering studies to improve TAG content and character in microalgae and ultimately contribute to the production of a sustainable liquid fuel.

## Methods

### Bioreactor experiments

*N. oleoabundans* (UTEX # 1185) was obtained from the Culture Collection of Algae at the University of Texas (UTEX, Austin, TX, USA). Batch cultures were started by inoculation with 10^6^ log growth phase cells into 1 liter glass flasks filled with 750 ml of Modified Bold-3 N medium [49] without soil extract. The concentration of nitrogen in the medium was adjusted to 50 mg as N l^-1^ (nitrogen replete; denoted as + N) and 10 mg as N l^-1^ (nitrogen limited; denoted as –N) using potassium nitrate (KNO_3_) as the sole source of nitrogen. These concentrations were chosen based on preliminary experiments that identified incubation times and nitrogen concentrations necessary to induce nitrogen depletion in the mid log-phase of the –N cultures and to ensure that the nitrogen-replete cultures never encountered nitrogen-limitation during the course of the experiment. For each nitrogen condition, cells were cultured in duplicate reactors. Reactors were operated at room temperature (25°C ± 2°C), and with a 14:10 h light:dark cycle of exposure to fluorescent light (32 Watt Ecolux, General Electric, Fairfield, CT, USA) at a photosynthetic photon flux density of 110 μmol-photon m^-2^ s^-1^. Cultures were mixed by an orbital shaker at 200 rpm and continuously aerated with sterile, activated carbon filtered air at a flow rate of 200 ml min^-1^ using a mass flow controller (Cole-Parmer Instrument Company, IL, USA).

### Nitrogen, biomass and biomolecule analysis

The nitrate concentration of culture media was determined daily by passage through a 0.2 μm pore-size filter and analysis on an ion chromatograph equipped with conductivity detection [50]. Microalgae growth was monitored daily by measuring the optical density of the cultures at 730 nm (OD_730_) using a spectrophotometer (HP 8453, Hewlett Packard, Palo Alto, CA, USA). Biomass samples for analysis of cellular constituents (starch, proteins, chlorophyll and lipids), and extraction of total RNA were harvested on day-11 by centrifugation at 10,000 *g* for 5 min at 4°C. Cell pellets were snap-frozen in liquid nitrogen and immediately transferred to −80°C until further analysis. The dry cell weight (DCW) of cultures was determined by filtering an aliquot of cultures on pre-weighed 0.45 μm pore size filters and drying the filters at 90°C until constant weight was reached. For analysis of starch content, 10^9^ cells ml^-1^ were suspended in deionized water in 2 ml screw-cap tubes containing 0.3 g of 0.5 mm glass beads, and disrupted by two cycles of bead-beating at 4800 oscillations per minute for 2 min, followed by three freeze/thaw cycles. The suspension was then incubated in a boiling water bath for 3 min and autoclaved for 1 hour at 121°C to convert starch granules into a colloidal solution. After samples were cooled to 60°C, cell debris was removed by centrifugation at 4,000 *g* for 5 min. The concentration of starch in the supernatant was measured enzymatically using the Sigma Starch Assay Kit (amylase/amyloglucosidase method, Sigma-Aldrich, Saint Louis, MO, USA) according to the manufacturer’s instruction. Chlorophyll a and b were measured by the N,N’-dimethylformamide method and calculated from spectrophotometric adsorption measurement at 603, 647, and 664 nm, as previously reported [51,52]. The total protein content of cells was determined with minor modifications to the original Bradford method [53] as described in [54]. Starch, chlorophyll, and protein measurements were performed in at least triplicates, and averages and standard deviations are reported as a percent of DCW.

The total lipid content of the cells was determined using a modified Bligh and Dyer method utilizing 2:1 chloroform:methanol [55]. To determine the profile of fatty acids, lipid samples were transesterified [56] and the resulting fatty acid methyl esters (FAME) were analyzed using a liquid chromatography-mass spectrometer (Varian 500-MS, 212-LC pumps, Agilent Technologies, Santa Clara, CA, USA) equipped with a Waters normal phase, Atlantis® HILIC silica column (2.1 × 150 mm, 3 μm pore size) (Waters, Milford, MA, USA), and atmospheric pressure chemical ionization [56]. Identification was based upon the retention time and the mass to charge ratio of standard FAME mixtures. The sum of FAME was used as a proxy for TAG content [22].

### RNA extraction, construction of cDNA libraries and DNA sequencing

To control for cell synchronization, cells for the + N and –N conditions were harvested at the same time of day. Total RNA was extracted and purified separately from each of the two nitrogen replete and the two nitrogen limited cultures using the RNeasy Lipid Tissue Mini Kit (Qiagen, Valencia, CA, USA). The quality of purified RNA was determined on an Agilent 2100 bioanalyzer (Agilent Technologies, Santa Clara, CA, USA). Isolation of mRNA from total RNA was carried out using two rounds of hybridization to Dynal oligo(dT) magnetic beads (Invitrogen, Carlsbad, CA, USA). Aliquots from mRNA samples were used for construction of the cDNA libraries using the mRNA-Seq Kit supplied by Illumina (Illumina, Inc., San Diego, CA, USA). Briefly, the mRNA was fragmented in the presence of divalent cations at 94°C, and subsequently converted into double stranded cDNA following the first- and second-strand cDNA synthesis using random hexamer primers. After polishing the ends of the cDNA using T4 DNA polymerase and Klenow DNA polymerase for 30 min at 20°C, a single adenine base was added to the 3’ ends of cDNA molecules. Illumina mRNA-Seq Kit specific adaptors were then ligated to cDNA 3’ ends. Next, the cDNA was PCR-amplified for 15 cycles, amplicons were purified (QIAquick PCR purification kit, Qiagen Inc., Valencia CA, USA), and the size and concentration of the cDNA libraries were determined on an Agilent 2100 bioanalyzer. Each of the four cDNA libraries (two nitrogen deplete and two nitrogen replete) was layered on a separate Illumina flow cell and sequenced at the Yale University Center for Genome Analysis using Illumina HiSeq 100 bp single-end sequencing. An additional lane was dedicated to sequencing PhiX control libraries to provide internal calibration and to optimize base calling. The sequence data produced in this study can be accessed at NCBI’s Sequence Read Achieve with the accession number SRA048723.

### RNA-seq data analyses

For quality control, raw sequencing reads were analyzed by FastQC tool (v0.10.0) [57] and low quality reads with a Phred score of less than 13 were removed using the SolexaQA package (v1.1) [58]. *De novo* transcriptome assembly was conducted using Velvet (v1.2.03) [23] and Oases (v0.2.06) [59] assembly algorithms with a multi-*k* hash length (i.e. 23, 33, 63, and 83 bp) based strategy to capture the most diverse assembly with improved specificity and sensitivity [59,60]. Final clustering of transcripts were obtained using the CD-HIT-EST package (v4.0-210-04-20) [61] and a non-redundant contigs set was generated.

For transcriptome annotation, the final set of contigs was searched against the NCBI’s non-redundant (nr) protein and plant refseq [24] databases using the BLASTX algorithm [62] with a cut off *E*-value ≤ 10^-6^. Contigs with significant matches were annotated using the Blast2GO platform [63]. Additional annotations were obtained through the Kyoto Encyclopedia of Genes and Genomes (KEGG) gene and protein families database through the KEGG Automatic Annotation Server (KAAS) (v1.6a) [64]. Associated Gene Ontology (GO) terms as well as enzyme commission (EC) numbers were retrieved and KEGG metabolic pathways were assigned [65].

To determine transcript abundances and differential expression, high quality reads from each experimental condition were individually mapped to the assembled transcriptome using Bowtie software (v0.12.7) [66]. Reads mapping to each contig were counted using SAMtools (v0.1.16) [67] and transcript abundances were calculated as reads per kilobase of exon model per million mapped reads (RPKM) [68]. All differential expression analysis (fold changes) and related statistical computations were conducted by feeding non-normalized read counts into the DESeq package (v1.5.1) [25]. Separate sequence read datasets were used as inputs into the DESeq package where size factors for each dataset were calculated and overall means and variances were determined based on a negative binomial distribution model. Fold change differences were considered significant when a q-value < 0.05 was achieved based on Benjamin and Hochberg’s false discovery rate (FDR) procedure [69], and only statistically significant fold changes were used in the results analysis. In addition to individual enzyme encoding transcripts, contigs were pooled for each experimental condition and tested against the combined dataset to determine the enriched GO terms using the Gossip package [70] integrated in the Blast2GO platform. Significantly enriched GO terms (q-value < 0.05) were determined for both + N and − N conditions.

Finally, reference guided mapping and differential expression was as also explored as a quantitation method. In this case, the Tophat package (v1.3.3) [71] was used to map high quality reads from each experimental condition against the genomes of closely related green algae species *Chlamydomonas reinhardtii* (version 169) and *Volvox carteri* (version 150) available through Phytozome (v7.0) [72]. Differential gene expression analysis was quantified using the Cufflinks package (v1.2.1) [73].

## Abbreviations

TAG: triacylglycerol; DCW: dry cell weight; ACCase: acytyl CoA carboxylase; ACP: acyl carrier protein; +N: nitrogen replete growth; -N: nitrogen-limited growth; FAME: fatty acid methyl ester; NCBI: national center for biotechnology information; GO: gene ontology; KEGG: Kyoto encyclopedia of genes and genomes; KO: KEGG orthology; EC: enzyme commission; HQ: high quality; NADPH: nicotinamide adenine dinucleotide phosphate; Log2FC: fold change of log2 transformed values; UTEX: the culture collection of algae at the University of Texas; RPKM: reads per kilobase exon model per million mapped reads; FDR: false discovery rate.

## Competing interests

The authors declare that they have no competing interests.

## Authors’ contributions

HR-Y carried out the growth experiments, conducted the transcriptome sequencing, and participated in the study design and in the preparation of the manuscript. BH assisted with the growth experiments and biomolecule measurements, performed the bioinformatics analysis, and assisted in the preparation of the manuscript. CH participated in the algal growth and biomolecule measurement. JP conceived the study, participated in the study design, and oversaw manuscript drafting. All authors read and approved the final manuscript.

## Authors' information

Hamid Rismani-Yazdi and Berat Z. Haznedaroglu denote equal authorship.

## Supplementary Material

Additional file 1 **Table containing*de novo*transcriptome assembly metrics for*N. oleoabundans.*** Click here for file

Additional file 2 **Table containing transcriptome annotation summary for*N. oleoabundans*.** Click here for file

Additional file 3 Complete spreadsheet of fold changes with significance levels for all transcripts assessed. Click here for file

Additional file 4 Table containing the complete list of broader GO terms and differential enrichment for the + N condition. Click here for file

Additional file 5 Table containing the complete list of broader GO terms and differential enrichment for the –N condition. Click here for file

Additional file 6 **Global pathway level representation of differential gene expression in*N. oleoabundans*.** Central metabolic pathways appear within the top right boxes and pathways associated with the biosynthesis of secondary metabolites are shown bottom right*.* Module level close-up representation (light gray boxes) of differential regulation are presented for (A) Fatty acid biosynthesis and metabolism, biosynthesis of unsaturated fatty acids, and glycerolipid metabolism; (B) Starch metabolism; (C) Nitrogen metabolism; (D) Terpenoid backbone synthesis; and (E) Diterpenoid and carotenoid biosynthesis. The metabolic pathway map was generated as described by Gianoulis et al. [24] using iPath2.0 [74]. Click here for file
