# Supplementary material for: Transcriptomic analysis of the oleaginous microalga Neochloris oleoabundans reveals metabolic insights into triacylglyceride accumulation
Source: Biotechnol Biofuels. 2012 Sep 24;5:74. doi: 10.1186/1754-6834-5-74 (PMC3549901; doi:10.1186/1754-6834-5-74)
Supplement: Additional file 1 — Table containingde novotranscriptome assembly metrics forN. oleoabundans. [file 1754-6834-5-74-S1.docx]

*De novo* transcriptome assembly metrics

|  | **Velvet-Oases** |
| --- | --- |
| **Sequencing** |  |
| Raw sequencing reads | 88,141,810  21,489,895  21,169,591  23,078,227  22,404,097  99  32 |
| -N Biological replicate-1 |  |
| -N Biological replicate-2 |  |
| +N Biological replicate-1 |  |
| +N Biological replicate-2 |  |
| Average read length |  |
| Average read quality |  |
| **Pre-assembly reads after QC and trimming** |  |
| Total pre-assembly reads | 87,090,842  20,992,976  20,865,479  22,975,146  22,257,241  77  35 |
| -N Biological replicate-1 |  |
| -N Biological replicate-2 |  |
| +N Biological replicate-1 |  |
| +N Biological replicate-2 |  |
| Average read length |  |
| Average read quality |  |
| **Assembly** |  |
| Number of transcripts | 56,550^*^ |
| Average length of transcripts | 1,459 |
| Longest transcript length | 14,688 |
| N50 | 2,561 |
| N90 | 776 |

^*^Following clustering and redundancy removal.
