# Supplementary material for: Transcriptomic analysis of the oleaginous microalga Neochloris oleoabundans reveals metabolic insights into triacylglyceride accumulation
Source: Biotechnol Biofuels. 2012 Sep 24;5:74. doi: 10.1186/1754-6834-5-74 (PMC3549901; doi:10.1186/1754-6834-5-74)
Supplement: Additional file 2 — Table containing transcriptome annotation summary forN. oleoabundans. [file 1754-6834-5-74-S2.docx]

Transcriptome annotation summary

|  |  |
| --- | --- |
| **BLAST** |  |
| Number of transcripts submitted for BLAST | 56,546 |
| Number of transcripts with a BLAST hit | 34,724 |
| Number of transcripts with functional mappings | 45,342 |
| Number of transcripts with Gene Ontology (GO) terms | 23,520 |
| Number of transcripts with enzyme commission (EC) numbers | 4,667 |
| Number of transcripts with KEGG ortholog (KO) numbers | 14,957 |

^*^Following clustering and redundancy removal.
