# Supplementary figures and images for: Transcriptomic analysis of the oleaginous microalga Neochloris oleoabundans reveals metabolic insights into triacylglyceride accumulation
Source: Biotechnol Biofuels. 2012 Sep 24;5:74. doi: 10.1186/1754-6834-5-74 (PMC3549901; doi:10.1186/1754-6834-5-74)

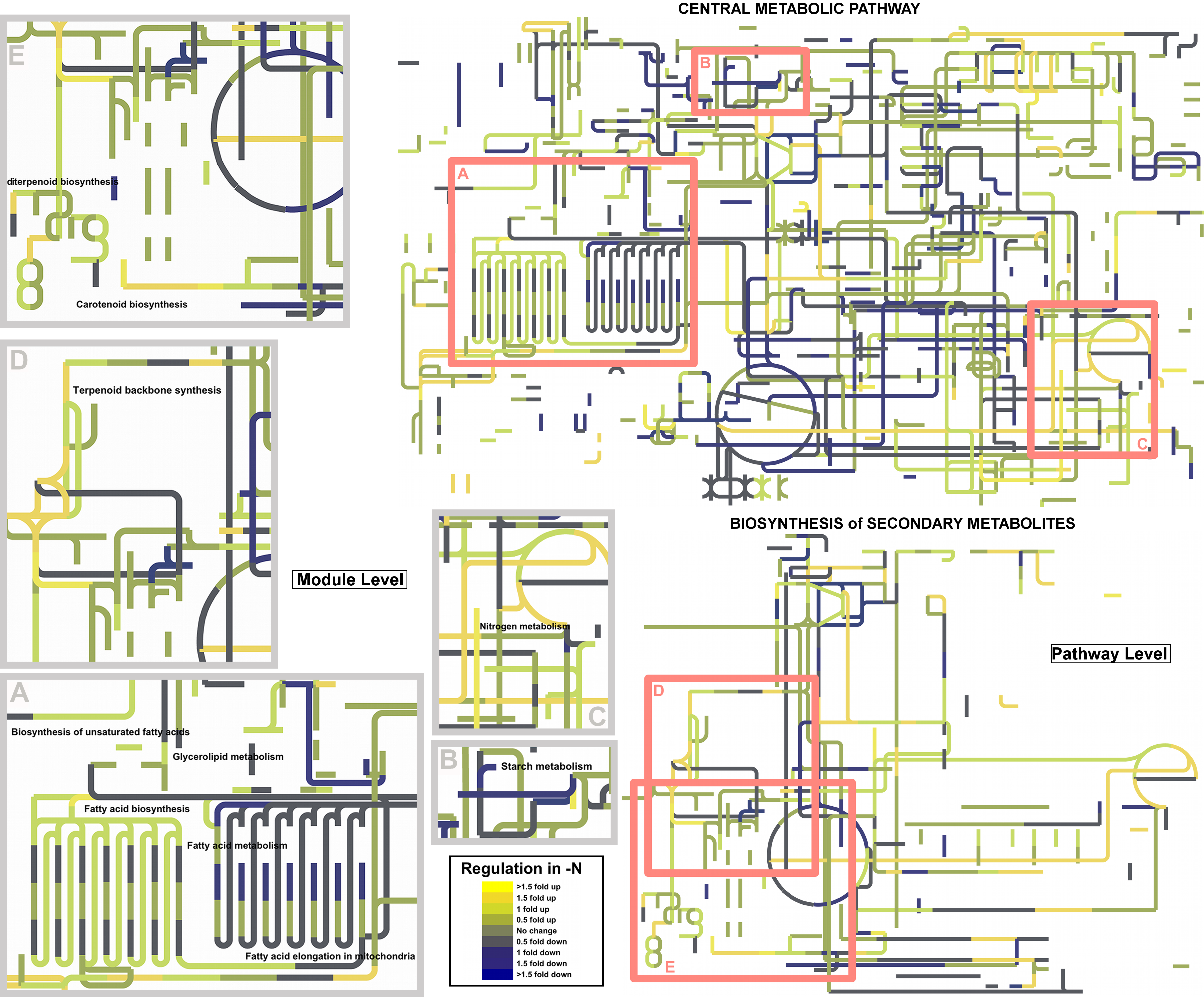

Supplement: Additional file 6 — Global pathway level representation of differential gene expression inN. oleoabundans. Central metabolic pathways appear within the top right boxes and pathways associated with the biosynthesis of secondary metabolites are shown bottom right. Module level close-up representation (light gray boxes) of differential regulation are presented for (A) Fatty acid biosynthesis and metabolism, biosynthesis of unsaturated fatty acids, and glycerolipid metabolism; (B) Starch metabolism; (C) Nitrogen metabolism; (D) Terpenoid backbone synthesis; and (E) Diterpenoid and carotenoid biosynthesis. The metabolic pathway map was generated as described by Gianoulis et al. [24] using iPath2.0 [74]. [file 1754-6834-5-74-S6.tiff]
